# Overexpression of *PtoMYB115* improves lignocellulose recalcitrance to enhance biomass digestibility and bioethanol yield by specifically regulating lignin biosynthesis in transgenic poplar

**DOI:** 10.1186/s13068-022-02218-7

**Published:** 2022-11-05

**Authors:** Chunfen Fan, Wenyi Zhang, YuHao Guo, Kuan Sun, Lijun Wang, Keming Luo

**Affiliations:** Chongqing Key Laboratory of Plant Resource Conservation and Germplasm Innovation, Integrative Science Center of Germplasm Creation in Western China (Chongqing) Science City, Key Laboratory of Eco-Environments of Three Gorges Reservoir Region, Ministry of Education, Institute of Resources Botany, School of Life Sciences, Southwest University, Chongqing, 400715 China

**Keywords:** MYB115, Lignin, Lignocellulose modification, Biomass saccharification, Bioethanol

## Abstract

**Background:**

Woody plants provide the most abundant biomass resource that is convertible for biofuels. Since lignin is a crucial recalcitrant factor against lignocellulose hydrolysis, genetic engineering of lignin biosynthesis is considered as a promising solution. Many MYB transcription factors have been identified to involve in the regulation of cell wall formation or phenylpropanoid pathway. In a previous study, we identified that *PtoMYB115* contributes to the regulation of proanthocyanidin pathway, however, little is known about its role in lignocellulose biosynthesis and biomass saccharification in poplar.

**Results:**

Here, we detected the changes of cell wall features and examined biomass enzymatic saccharification for bioethanol production under various chemical pretreatments in *PtoMYB115* transgenic plants. We reported that *PtoMYB115* might specifically regulate lignin biosynthesis to affect xylem development. Overexpression of *PtoMYB115* altered lignin biosynthetic gene expression, resulting in reduced lignin deposition, raised S/G and beta-*O*-4 linkage, resulting in a significant reduction in cellulase adsorption with lignin and an increment in cellulose accessibility. These alterations consequently improved lignocellulose recalcitrance for significantly enhanced biomass saccharification and bioethanol yield in the *PtoMYB115*-OE transgenic lines. In contrast, the knockout of *PtoMYB115* by CRISPR/Cas9 showed reduced woody utilization under various chemical pretreatments.

**Conclusions:**

This study shows that *PtoMYB115* plays an important role in specifically regulating lignin biosynthesis and improving lignocellulose features. The enhanced biomass saccharification and bioethanol yield in the *PtoMYB115*-OE lines suggests that *PtoMYB115* is a candidate gene for genetic modification to facilitate the utilization of biomass.

**Supplementary Information:**

The online version contains supplementary material available at 10.1186/s13068-022-02218-7.

## Background

Wood, as an abundant, green and renewable resource, has been widely used for timber products, paper pulping, biochemicals and biofuels [1, 2]. Wood is structured mainly with thickened secondary cell walls, which primarily consist of cellulose impregnated with hemicelluloses and lignin. For effective utilization of wood, it is necessary to overcome the recalcitrance in pulping and biofuels production. Being a characteristic component of wood, lignin is considered as a major contributor to lignocellulose recalcitrance [3]. In higher plants, lignin is derived from the carbon skeleton of phenylalanine by sequential conversion and complex polymerization, and is an aromatic heteropolymer primarily consisting of guaiacyl (G), syringyl (S), and *p*-hydroxyphenyl (H) unit [4]. Different types of lignin monomer bonds are found in lignin polymers, such as β-*O*-4, β–β, α-*O*-4, 4-*O*-5, β-5, etc. [5, 6]. In the process of enzymatic saccharification of lignocellulose, lignin does not only act as a physical barrier to hinder the accessibility of enzymes to carbohydrates, but also restricts enzymatic hydrolysis through non-productive adsorption of enzymes [7, 8]. The lignin-based derivatives and lignin macromolecule also has negative influence on subsequent enzymatic hydrolysis [9]. In addition to lignin content, the structure and the linkages type of lignin monomers, are also considered as the key factors determining the enzymatic hydrolysis of lignocellulose [10–13]. In general, higher S or S/G ratio is accompanied with a higher proportion of β-*O*-4 linkages, which facilitate subsequent depolymerization and enzymatic saccharification [14, 15].

To overcome the recalcitrance of lignocellulose in secondary cell wall, harsh physical and chemical pretreatments are used by removing lignin, which demands high energy input, leading to potential secondary chemical pollution in the environment. In the past decades, a number of studies have been reported to realize the engineering of lignin by reducing the lignin content or changing the composition of lignin monomers. For example, down-regulation of the coding genes of key enzymes in the monolignol biosynthetic pathways, such as caffeic acid 3-*O*-methyltransferase (COMT) [16, 17], hydroxycinnamoyl-CoA shikimate hydroxycinnamoyl transferase (HCT) [18], cinnamoyl-CoA reductase (CCR) [19], ferulate 5-hydroxylase (F5H) [20–22], caffeoyl shikimate esterase (CSE) [23], cinnamyl alcohol dehydrogenase (CAD) [24], laccases for monolignol polymerization [25], and feruloyl-CoA monolignol transferase (FMT) [26], to alter lignin content, composition or ester linkages, thus resulting in reducing biomass recalcitrance and enhancing cell wall digestibility.

During the formation of secondary cell wall, lignin deposition is tightly orchestrated by a series of transcription factors (TFs) that dynamically regulate the expression of lignin biosynthetic genes [27–30]. Among these TFs, many MYBs (v-myb avian myeloblastosis viral oncogene homolog) are shown to regulate multiple branches of the phenylpropanoid biosynthetic pathway [31–41]. Compared with the limited secondary growth in *Arabidopsis* or grasses, perennial trees had continuous secondary growth in stems [42]. Therefore, it is still necessary to further understanding of the dynamic regulation of xylem lignification in tree species.

In previous studies, a poplar R2R3-MYB transcriptional factor PtoMYB115 has been identified to be involved in the regulation of proanthocyanidin biosynthesis and enhancing fungal resistance [43, 44]. Overexpression of *PtoMYB115* also down-regulated the expression of the lignin biosynthetic genes [43], however, it is unclear whether *PtoMYB115* regulates secondary wall formation, especially its impact on lignocellulose properties and biomass glycosylation in bioethanol production. Here, we characterized the role of *PtoMYB115* in cell wall formation during wood development and evaluated its effect on biomass enzymatic saccharification and bioethanol production in poplar.

## Results

### *PtoMYB115* represses xylem development and cell wall biosynthesis

**Fig. 1:**
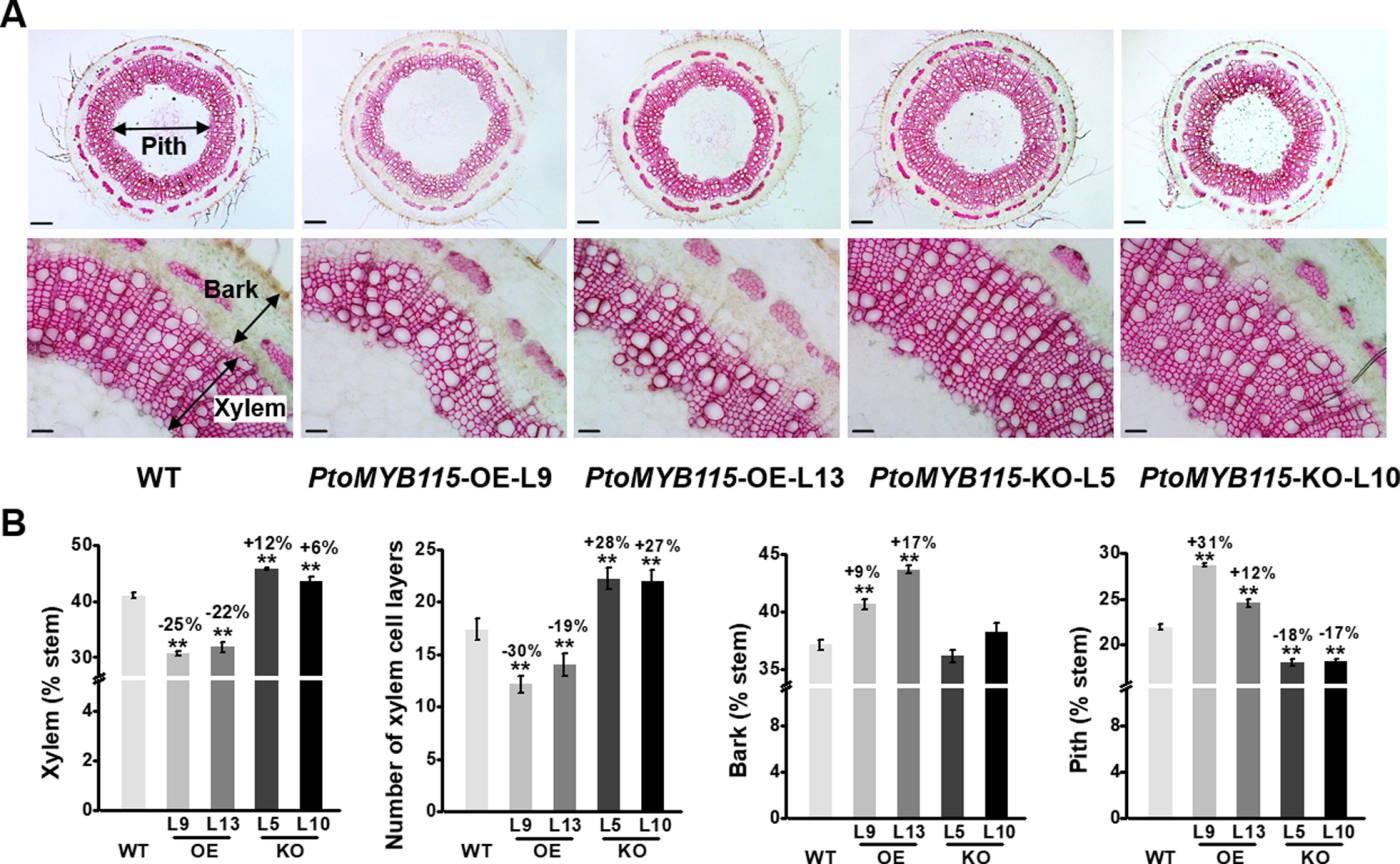
Observations of wood formation in the *PtoMYB115* transgenic lines and WT. **a** Phloroglucinol–HCl staining of stem cross sections in 6th internode stems of 3-month-old transgenic lines and WT (scale bars as 200 μm and 100 μm, respectively). **b** Numbers of xylem cell layers (cell files) and lumen area of individual xylem vessel cell and fiber cell. All data as means ± SD (*n* = 30). Increased percentage (%) obtained by subtracting transgenic line value with WT divided by WT. Student’s *t*-test was performed between the transgenic line and WT as **P < 0.01

To investigate the function of *PtoMYB115* in growth and development of poplar, the *PtoMYB115*-overexpressed (OE) and -knockout (KO) mutant lines using the CRISPR/Cas9 system were regenerated as described previously [43] (Additional file 1: Fig. S1). We observed stem morphology and determined cell wall composition in these transgenic plants (Fig. 1). Compared with wild-type (WT) plants, the *PtoMYB115*-OE lines exhibited significantly reduction in xylem development, while the *PtoMYB115*-KO lines showed the opposite phenotype (Fig. 1A). Quantitative analysis revealed that the xylem area and xylem cell layers of the *PtoMYB115*-OE lines were significantly decreased, but the bark and pith area was increased, in comparison with the WT (Fig. 1B). In contrast, increased xylem cell layers and less pith area were observed in the stems of the *PtoMYB115*-KO lines (Fig. 1B). Additionally, the expression of the genes *PtoLBD38* and *PtoCLE14*, which are involved in the regulation of xylem cell differentiation or stem cell activity [45], was down-regulated in the *PtoMYB115*-OE lines but upregulated in the *PtoMYB115*-KO lines, compared with the WT (Additional file 1: Fig. S2).

**Fig. 2:**
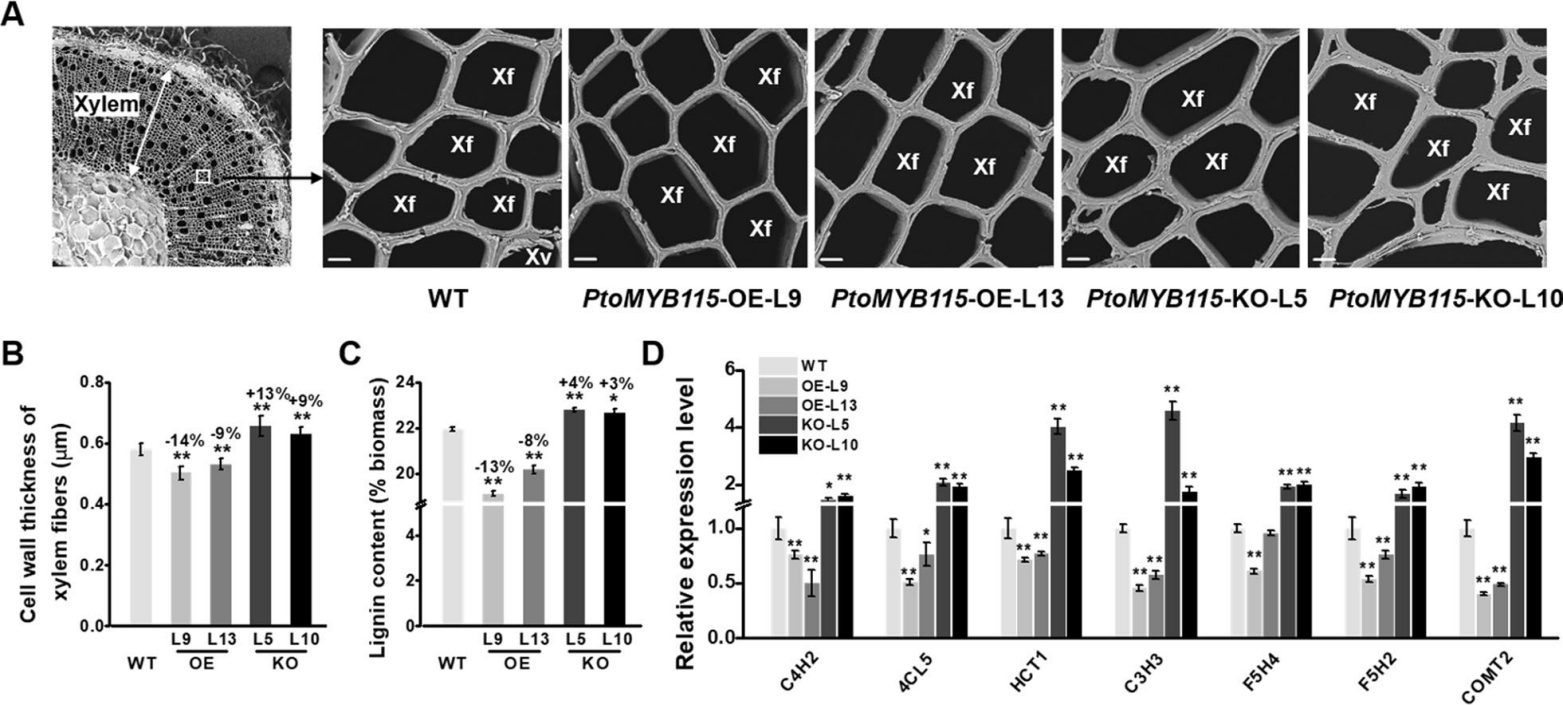
Observations of plant cell wall formation in the *PtoMYB115* transgenic lines and WT. **a** Scanning electron microscopy (SEM) images (*Xv* xylem vessel cells, *Xf* xylem fiber cells, scale bars as 10 μm). **b** Cell wall thickness of SEM observation in xylem fiber cells. **c** Lignin content (% biomass). **d** Expression of genes involved in lignin biosynthesis. All data as means ± SD. Student’s *t*-test was performed between the transgenic line and WT as *P < 0.05 and **P < 0.01 (*n* = 30 in b, *n* = 3 in **c** and **d**)

We further observed the cell wall thickness of the *PtoMYB115*-OE and *PtoMYB115*-KO lines by scanning electron microscopy (SEM). As shown in Fig. 2A, B, we observed that cell wall thickness of the stems in the *PtoMYB115*-OE lines were significantly reduced (*P* < 0.01), while that of the *PtoMYB115*-KO lines were increased, compared to the WT. A chemical analysis of cell wall showed that, in comparison to that of WT plants, lignin content in the stems in two *PtoMYB115*-OE lines (L9 and L13) were decreased by 13% and 8%, while that of *PtoMYB115*-KO lines (L5 and L7) were increased by 4% and 3%, respectively (Fig. 2C). Accordingly, real-time quantitative PCR (RT-qPCR) analysis revealed that the expression of these lignin biosynthetic genes, including *C4H2*, *4CL5*, *HCT1*, *C3H3*, *F5H4*, *F5H2*, and *COMT2* [4, 22], was drastically down-regulated in the *PtoMYB115*-OE lines, but significantly elevated in the *PtoMYB115*-KO lines (Fig. 2D). In contrast, other two major wall polymers (cellulose and hemicellulose) and genes involved in cellulose and hemicelluloses biosynthesis were not significantly altered in all transgenic lines (Additional file 1: Fig. S2). Taken together, these results suggest that PtoMYB115 may be involved in the regulation of lignin biosynthesis in secondary cell wall, and affecting xylem development in poplar.

### *PtoMYB115* affected lignin composition and linkage patterns

**Fig. 3:**
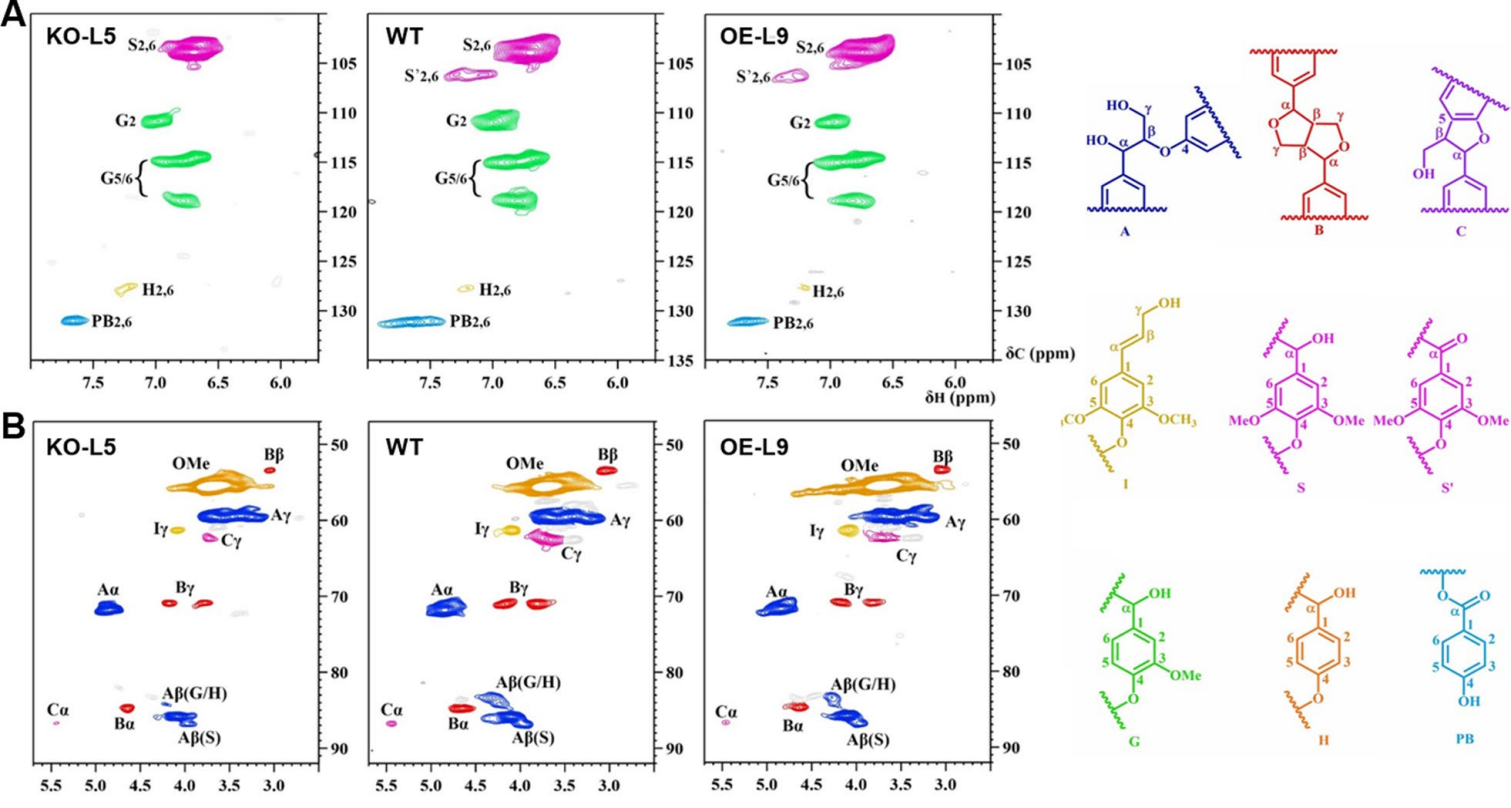
2D HSQC NMR spectral analysis of lignin structure in the *PtoMYB115* transgenic lines and WT. **a** Lignin aromatic region is shown for *PtoMYB115*-KO-L5, WT, and *PtoMYB115*-OE-L9. Correlations from the various aromatic ring unit types are well dispersed and can be categorized as the core lignin units (S syringyl, G guaiacyl, H *p*-hydroxyphenyl). **b** The lignin side chain region is shown for *PtoMYB115*-KO-L5, WT, and *PtoMYB115*-OE-L9. The major inter-unit structural units are β-ether (β-*O*-4, **a**), phenylcoumaran (β-5, **b**), and resinol (β–β, **c**), color-coded by their indicated structures

As the lignin contents were altered in transgenic plants, we further evaluate the lignin structure and composition by solution-state two-dimensional (2D) _1_H–_13_C correlation HSQC (gradient-enhanced heteronuclear single-quantum coherence) NMR analysis. The changes in lignin monomer composition, the bonding types and distribution of inter-unit linkage patterns were readily visualized [46, 47]. As shown in Fig. 3, in the aromatic regions of the lignin, typical G and S units were detected, while only trace level of H lignin was observed in all the woody samples. Compared to WT, higher level of S unit was detected in *PtoMYB115*-OE transgenic line, resulting in an increased S/G ratio (Table 1). In contrast, decreased S unit and S/G ratio were detected in *PtoMYB115*-KO transgenic line (Table 1). In addition, uncommon syringyl (S′) units derived from polymerization of sinapyl aldehyde were present in WT and *PtoMYB115*-OE transgenic line, but absent in *PtoMYB115*-KO line. For the aliphatic regions of lignin in the spectra, typical correlation peaks from the conspicuous methoxyl groups (OMe) and the substructures, such as β-ether (β-*O*-4, A), phenylcoumaran (β-5, B), and resinol (β–β, C) units were identified. Semiquantitative analysis of the 2D HSQC peaks was performed for the relative percentage estimation. The relative content of β-*O*-4 linkage was 66.26/100 Ar in WT, with slightly increased to 69.68/100 Ar in the *PtoMYB115*-OE transgenic line. In contrast, *PtoMYB115*-KO transgenic line had reduced β-*O*-4 linkages (Fig. 3, Table 1). These results suggest that PtoMYB115 also participated in the regulation of monolignol biosynthesis (the S/G ratio) and the lignin linkage patterns.

**Table 1:** Quantification of lignin fractions by quantitative 2D-HSQC NMR (results expressed per 100 Ar)

Sample	β-*O*-4	β–β	PB	S/G	S/G/H
*PtoMYB115-*KO-L5	62.29	11.67	6.28	1.94	64/33/3
WT	66.26	12.80	7.84	2.63	71/27/2
*PtoMYB115-*OE-L9	69.68	14.63	6.61	2.92	73/25/2

### *PtoMYB115* enhanced biomass enzymatic saccharification and bioethanol yield

**Fig. 4:**
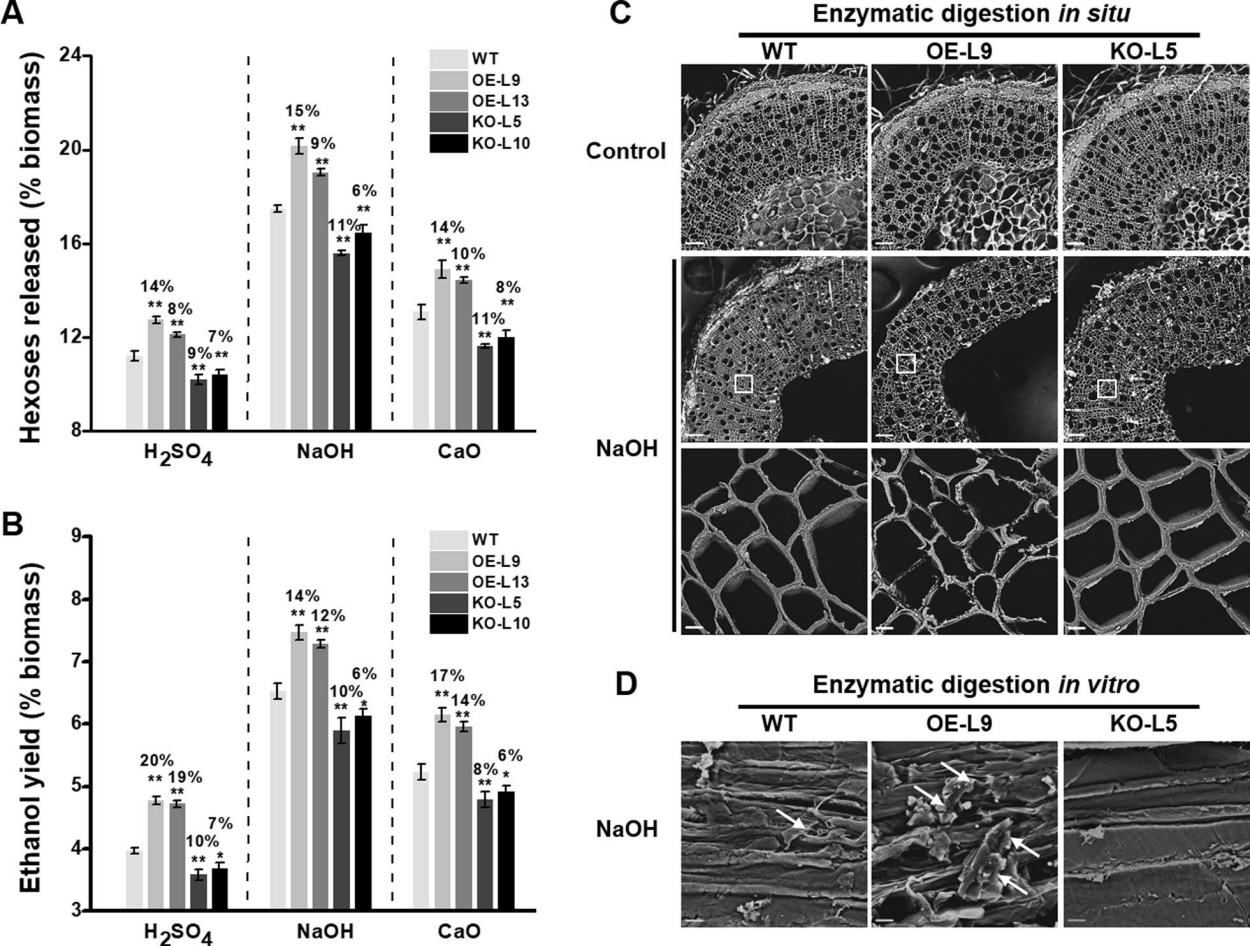
Analyses of biomass enzymatic saccharification in the *PtoMYB115* transgenic lines and WT. **a** Hexose yields (% biomass) released from enzymatic hydrolysis after the pretreatment with 4% H_2_SO_4_, 4% NaOH, or 10% CaO pretreatments. **b** Bioethanol yields (% biomass) obtained from yeast fermentation using total hexose contents released from enzymatic hydrolysis after pretreatments. **c** SEM images of in situ enzymatic digestion of stem cross sections after 4% NaOH pretreatment and sequential enzymatic hydrolysis, scale bar is 100 μm or 5 μm; **d** SEM images of biomass residue obtained from 4% NaOH pretreatment and sequential enzymatic hydrolysis. Allows as rough point. Scale bar is 5 μm. Data represent mean ± SD of three technical replicates. Student’s *t*-test between transgenic line and WT as **P < 0.01

As lignin has been considered as one of the crucial factors negatively affecting biomass digestibility [3, 7–9], we next measured the hexoses yields released from enzymatic hydrolysis in WT and transgenic plants. Using our previously established approaches [48], three mild acid and alkali pretreatments (4% H_2_SO_4_/120 ºC/20 min; 4% NaOH/50 ºC/2 h; and 10% CaO/50 ºC/2 h) were used in this study. Obviously, the NaOH pretreatment caused the highest hexoses yields in both transgenic lines and WT, among these pretreatments. In detail, the pretreatment with H_2_SO_4_, NaOH or CaO could lead to hexoses yields (% biomass) 8–14%, 9–15%, or 10–14% increased from *PtoMYB115*-OE lines than the WT, respectively, while the *PtoMYB115*-KO transgenic lines had reduced hexoses yields after these pretreatments (Fig. 4A). In addition, this study performed a classic yeast fermentation using total hexoses released from enzymatic hydrolysis [48]. Under these pretreatments, the *PtoMYB115*-OE lines showed significantly higher bioethanol yields (% biomass) than those of the WT at *P* < 0.01 level. In detail, the pretreatment with H_2_SO_4_, NaOH or CaO could lead to bioethanol yields 19–20%, 12–14% or 14–17% increased from *PtoMYB115*-OE lines than the WT (Fig. 4B), respectively. Such large enhancements were confirmed by SEM visualizations of more violent destruction of stem tissue in situ (Fig. 4C), and of rougher biomass residue surfaces in *vitro* (Fig. 4D) in the *PtoMYB115*-OE lines. Conversely, knockout of *PtoMYB115* led to much lower bioethanol yields compared with the WT. Hence, these results indicate that, under the relatively cost-effective pretreatments, overexpression of *PtoMYB115* lead to the continuous improvement of biomass enzyme saccharification and bioethanol productivity.

### Reduced cellulase adsorption for high biomass saccharification in the *PtoMYB115*-OE lines

**Fig. 5:**
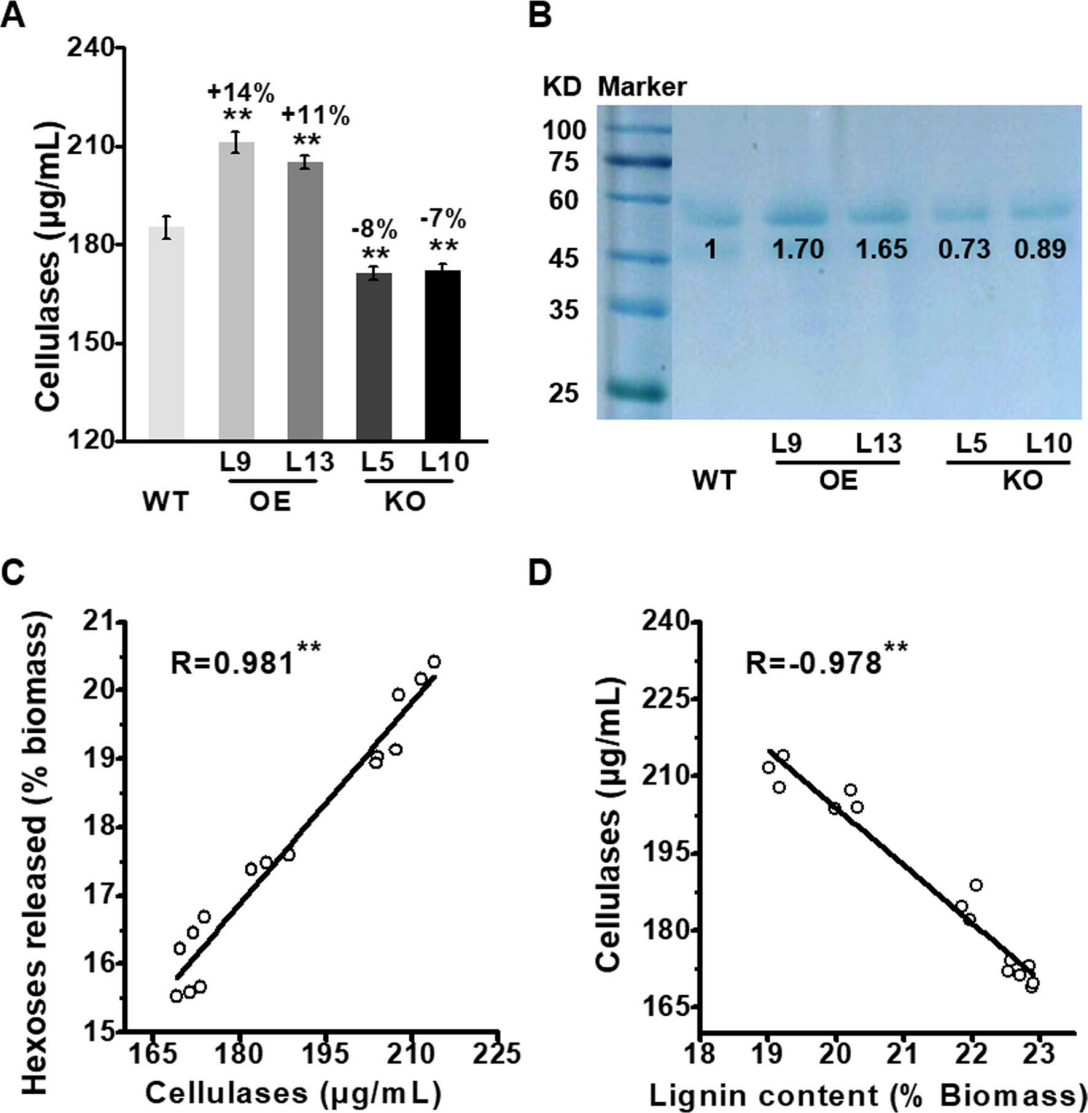
Cellulase enzyme adsorption in *PtoMYB115* transgenic lines and WT. **a** Total soluble enzyme levels (μg/mL) of the supernatant collected from enzymatic hydrolysis; **b** SDS-PAGE profiling of the soluble enzymes; **c** correlation analysis between the cellulase contents and hexose yields (% biomass) released from enzymatic hydrolyses of lignocellulose substrates examined; **d** correlation analysis between the cellulase content and lignin contents (% biomass). **Significant correlation at *P* < 0.01 (*n* = 3 in a, *n* = 15 in **c**, **d**)

Since lignin content and structure were defined to account for cellulase enzyme loading and adsorption access to cellulose microfibrils [7–13], we further examined the soluble protein contents in the supernatants of enzymatic hydrolyses with the pretreated biomass residues (Fig. 5). Compared to the WT, the *PtoMYB115*-OE lines contained significantly increased soluble proteins at *P* < 0.01, with the protein levels increased by 11–14% (Fig. 5A). SDS-PAGE profiling (Fig. 5B) also showed that the soluble proteins, which were derived from the commercial mix-cellulases incubated with the pretreated biomass residues for enzymatic hydrolysis, were higher in *PtoMYB115*-OE lines. In addition, the soluble proteins levels revealed a significantly positive correlation with the hexose yields released from enzymatic hydrolysis (*n* = 15, *R* = 0.981; Fig. 5C), indicating that the *PtoMYB115*-OE lines with increased biomass saccharification is partially due to the reduced adsorption of mixed cellulase with the pretreated biomass residues. Furthermore, the soluble protein levels were negatively correlated with lignin contents of the biomass residues (*n* = 15, *R* = − 0.978; Fig. 5D), suggesting that lignin may be specific for enzyme adsorption. Taken together, these results suggest that the reduction of lignin content could inhibit cellulase adsorption, leading to an integrated enhancement of its biomass enzymatic saccharification in the *PtoMYB115*-OE lines.

### Altered cellulose features and cellulose accessibility in *PtoMYB115* transgenic poplar

**Fig. 6:**
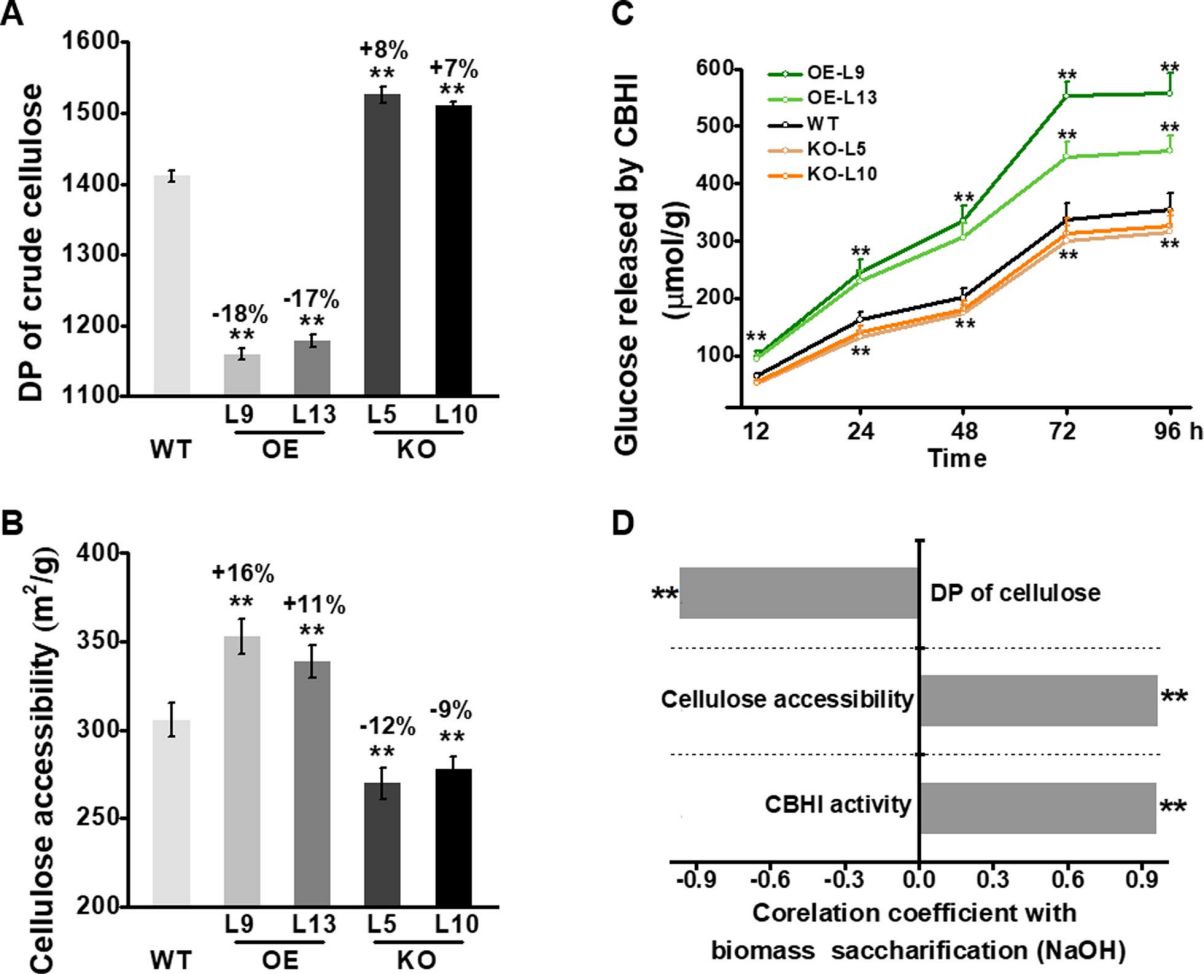
Comparison of lignocellulose features between the *PtoMYB115* transgenic lines and WT. **a** Degree of polymerization (DP) of crude cellulose; **b** cellulose accessibility by measuring Congo red (CR) dye area; **c** glucose yield of the cellobiose released from time-course CBHI hydrolyzes using crude cellulose as substrate; **d** correlation analysis between lignocellulose features and hexose yields (% biomass) released from enzymatic hydrolyses after NaOH pretreatment. All data are means ± SD of three technical replicates. **Significant correlation at *P* < 0.01 (*n* = 3 in **a**–**c**, *n* = 15 in **d**)

It has been demonstrated that lignocellulose features significantly affect biomass enzymatic saccharification under various pretreatments [37]. Due to the marked changes in lignin features and biomass saccharification in *PtoMYB115* transgenic poplar, we subsequently examined other lignocellulose features. Compared to the WT, the *PtoMYB115*-OE lines showed reduced degree of polymerization (DP) values by 17–18%, while the *PtoMYB115*-KO lines showed increased DP (Fig. 6A). But no significant difference of cellulose crystalline index (CrI) was measured between *PtoMYB115* transgenic lines and WT (Additional file 1: Fig. S3A). As the cellulose accessibility of the pretreated residues is the direct parameter accounting for cellulase enzyme attack on cellulose surface [49], we measured the cellulose accessibility in WT and transgenic plants. By comparison, the pretreated residues of the *PtoMYB115*-OE lines showed significantly (*P* < 0.01) increased cellulose accessibility by 11–16% than that of the WT (Fig. 6B), suggesting that the *PtoMYB115*-OE lines had more cellulase loading and attacking surface sites. This result was supported by other findings as follows: in the process of enzymatic hydrolysis, the glucose yields released by the cellobiohydrolase (CBH I) hydrolyses in the *PtoMYB115*-OE lines are 1.5 times higher than that in the WT (Fig. 6C). Because CBHI specifically attacks the reducing-ends of β-1,4-glucan chains to produce cellobiose [50], this result could directly explain the increase of cellulose accessibility in the *PtoMYB115*-OE lines. Conversely, *PtoMYB115*-KO transgenic lines showed reduced cellulose accessibility and CBHI activity (Fig. 6A–C). Additionally, we detected that the DP of cellulose was negatively correlated with the biomass enzymatic saccharification under various pretreatments (*R*^*2*^ > 0.9) in the transgenic plants (Fig. 6D, Additional file 1: Fig. S3B, C). Notably, we also found that the cellulose accessibility and CBHI activity were positively correlated with biomass enzymatic saccharification (*R*^*2*^ > 0.9) (Fig. 6D, Additional file 1: Fig. S3B, C). These results indicate that the reduced DP and increased cellulose accessibility might result in enhanced biomass enzymatic saccharification in the *PtoMYB115*-OE plants.

## Discussion

In previous studies, PtoMYB115 has been shown to be involved in regulating proanthocyanidin biosynthesis [43, 44]. However, it remains to explore whether PtoMYB115 regulates wood development and lignocellulose biosynthesis. In this study, overexpression of *PtoMYB115* showed much reduced xylem development with decreased xylem cell layers and cell wall thickness, while knockout of *PtoMYB115* has expanded xylem area and thicker cell wall (Figs. 1–2). Accordingly, overexpression of *PtoMYB115* can repress the expression of genes related to lignin biosynthesis, leading to a significant decrease in lignin level, while knockout of *PtoMYB115* showed increased lignin content (Fig. 2). By comparison, no significant impacts on other two major wall polymers (cellulose, hemicellulose) were detected in all transgenic lines (Additional file 1: Fig. S2). These results support that *PtoMYB115*, as a negative regulator, specially regulates lignin deposition during wood formation. In a previous study, we observed that most of the structural enzyme genes in flavonoids pathway (*CHS4*, *CHI*, *ANS2*, *DFR1*, *ANR1*, *LAR1* and *LAR3*) were upregulated in the *PtoMYB115*-OE plants compared with the WT [43]. Similarly, we also demonstrated that *PtoMYB115* activated the promoters of the flavonoid biosynthetic genes (*CHS4*, *DFR1*, *ANR1* and *LAR3*). Taken together, we speculate that the *PtoMYB115* is critical for the regulation of different branches (lignin and flavonoids) in phenylpropanoid biosynthetic pathways.

In poplar, most of the MYB proteins are activators that positively regulate cell wall biosynthesis, less MYB genes are reported as repressors [38–42]. Previous studies have shown that *PtoMYB115* is a transcriptional activator in yeast [43], and interacts with PtoKNAT7 [29]. In *Arabidopsis*, AtKNAT7 is a negative regulator of secondary wall biosynthesis [51]. It would be worth investigatin the function of PtoKNAT7 on secondary cell wall biosynthesis, and whether *PtoMYB115* represses lignin metabolism by interacting with PtoKNAT7 in poplar.

In terms of much altered lignin levels in transgenic lines, this study also examined the other major lignocellulose features in transgenic poplar. Compared to the WT, the *PtoMYB115*-OE transgenic lines showed higher level of S but less G lignin, resulting in an increased S/G ratio. The relative β-*O*-4 linkage was also increased in the *PtoMYB115*-OE lines (Fig. 3, Table 1). Subsequently, higher soluble cellulase content with reduced cellulase adsorption were observed in the *PtoMYB115*-OE lines (Fig. 5). In contrast, decreased S, S/G ratio, β-*O*-4 linkage (Fig. 3, Table 1) and cellulase content (Fig. 5) were detected in the *PtoMYB115*-KO lines. Besides the lignin features mentioned above, cellulose features were also altered. The *PtoMYB115*-OE lines had significantly reduced cellulose DP values (Fig. 6A), as well as increased cellulose accessibility and CBHI activity (Fig. 6 B, C). Conversely, the *PtoMYB115*-KO lines showed increased DP, and reduced cellulose accessibility (Fig. 6). In plant cell wall, lignin is branched molecules crosslinking the hemicelluloses and possibly even hemicelluloses to cellulose fibrils [3, 10]. These major components are cross-linked to form lignin–carbohydrate complexes [13]. There is no difference in cellulose content and the expression of cellulose synthases among all transgenic plants (Additional file 1: Fig. S2). Thus, we speculate that the changes of cellulose DP and cellulose accessibility are probably due to altering lignin features and the cross link between lignin and wall polysaccharides in the *PtoMYB115* transgenic lines. The features of modified lignin (reduced lignin deposition, raised S/G ratio and β-*O*-4 linkage) should be able to effectively extract lignin, therefore reducing cellulose DP and increasing cellulose accessibility in the *PtoMYB115*-OE lines. It has been reported that reducing lignin content, changing the composition or ester linkages of lignin monomers can improve cell wall digestibility [16–26]. However, little is yet reported about the impacts on cellulose and hemicellulose features in these lignin-altered plants. Therefore, it remains to be explored whether it is used to regulate the biosynthesis of other cell wall polymers and how lignocellulose properties are mediated.

Since the composition and basic features of plant cell wall determine the biomass saccharification [3, 7–15, 37], our study examined the hexoses yields released from enzymatic hydrolysis after various chemical pretreatments, and the bioethanol yield by yeast fermentation using hexoses released from enzymatic hydrolysis. In general, the *PtoMYB115*-OE lines produced much higher hexoses and bioethanol yields, but the *PtoMYB115*-KO lines had reduced hexoses and bioethanol yields (Fig. 4). Then correlation analyses were applied to account for wall polymer impacts on biomass enzymatic saccharification (Figs. 5, 6). The level of soluble cellulase is positively correlated with the yield of hexose released by enzymatic hydrolysis, but lignin contents were negatively correlated with the level of soluble protein (Fig. 5), indicating that lignin may be a specific substance for enzyme adsorption as reported by previous studies [7, 8]. The DP of cellulose was negatively correlated with the biomass enzymatic saccharification, but the cellulose accessibility and CBHI activity were positively correlated with biomass enzymatic saccharification (Fig. 6), which confirms that the DP, cellulose accessibility and CBHI activity are key factors for biomass enzymatic digestibility [50, 52–54].

**Fig. 7:**
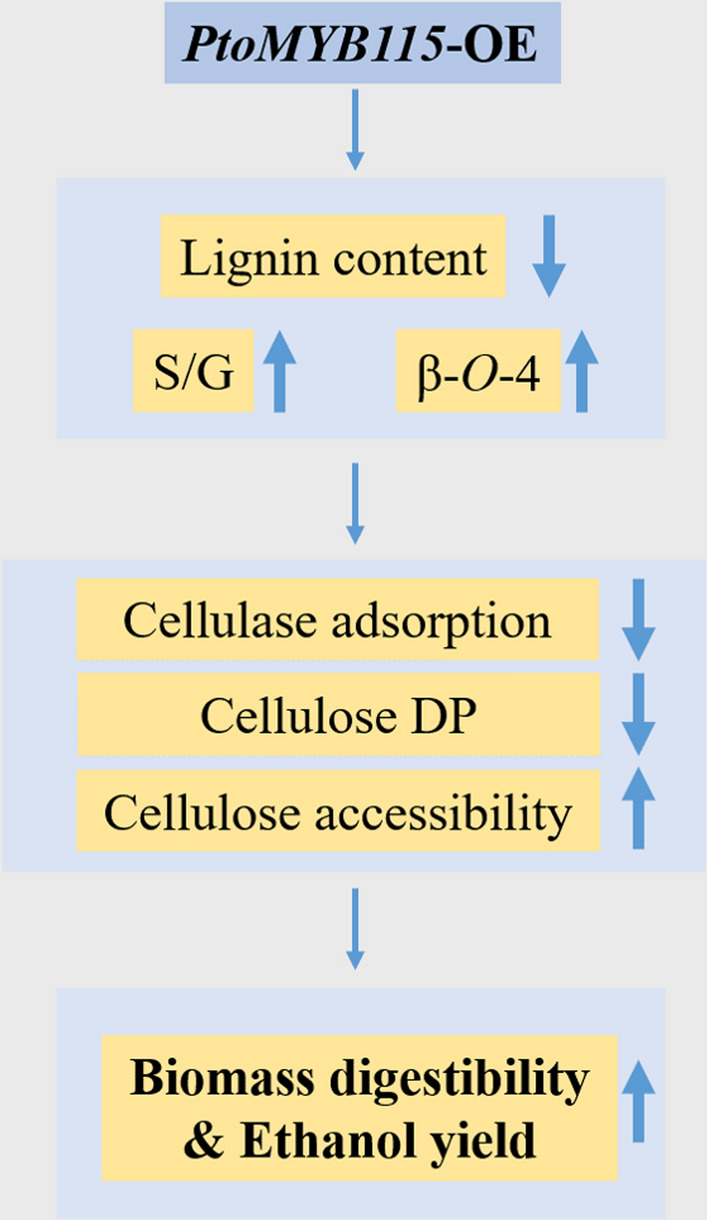
A hypothetical model to demonstrate an integrated approach effective for high bioethanol production in lignocellulose-improved *PtoMYB115*-OE transgenic poplar plants

Based on these results obtained from our study, as shown in Fig. 7, a tentative model is proposed to explain why the biomass saccharification for the higher bioethanol production was increased in the *PtoMYB115*-OE transgenic plants: (1) *PtoMYB115* altered the expression of genes involved in lignin biosynthesis, results in reduced lignin content and raised S/G and beta-*O*-4 linkage; (2) the reduction of lignin level not only weakens the physical barrier of enzymes to carbohydrates, but also decreases the unproductive adsorption of enzymes to lignin, improving cellulose accessibility, so that more cellulose enzymes act on the cellulose surface of the *PtoMYB115*-OE lines; (3) the structural modification in lignin (reduced lignin content, increased S, S/G ratios, and β-*O*-4 lignin linkage) may loosen the interactions between lignin and carbohydrates, causing lower cellulose DP to be detected during lignocellulose sampling for preparing crude or crystalline cellulose substrates in the *PtoMYB115*-OE lines; (4) the reduced cellulose DP improves the reducing-ends of cellulose chains, and the increase of cellulose accessibility are positive for the access and loading of celluloses, therefore leading to the remarkable increase of biomass enzymatic saccharification and high ethanol conversion in the *PtoMYB115*-OE plants.

## Conclusion

This study shows that *PtoMYB115* specifically regulates lignin biosynthesis in the process of xylem lignification in poplar. Overexpression of *PtoMYB115* can significantly suppress lignin content, increase lignin S/G ratio, and β-*O*-4 lignin linkage, which thereby result in reduced cellulose DP, and increased biomass accessibility. These changes of lignocellulose features consequently improve lignocellulose recalcitrance, leading to remarkably enhanced biomass enzymatic saccharification and bioethanol yield under green-like and cost-effective chemical pretreatments. Hence, our study provides a powerful strategy for genetic modification of cell walls and low-cost utilization of lignocellulose in plants.

## Materials and methods

### Experimental procedures

#### Plant materials and transformation

The *PtoMYB115* cDNA was cloned from *Populus tomentosa*, and inserted into the pCXSN vector after 35S promoter. Three CRISPR/Cas9 target sites of *PtoMYB115* were assembled into binary pYLCRIPSR/Cas9 vector. Genetic transformation of *P. tomentosa* was performed via *Agrobacterium*-mediated infiltration of leaf disks. The positive transgenic lines were selected based on the hygromycin selection and PCR analysis [43]. All primers used are listed in Additional file 1: Table S1.

#### RNA extraction and RT-qPCR

Total RNA was extracted from the stems of 3-month-old poplar trees using a Plant Total RNA Extraction Kit (Bioflux, China). Complementary DNA was synthesized using a PrimeScript RT reagent kit with gDNA Eraser (0047A, TAKARA, Dalian, China). RT-qPCR was performed using Hieff qPCR SYBR Green Master Mix (11201ES08, YEASEN, China) according to the manufacturer’s instructions in a qTOWER3G IVD Real-Time PCR machine (Analytik Jena AG, Germany). The poplar *UBIQUITIN* gene was used as the reference gene. The primers are listed in Additional file 1: Table S1.

#### Microscopic observation

The sixth internode of the 3-month-old poplar stems were fixed in FAA buffer (formaldehyde:glacialacetic acid:50% ethanol, 1:1:18). After embedding in paraffin, the stems were cross sectioned by using an Ultra-Thin Semiautomatic Microtome (FINESSE 325, Thermo). For lignin staining, sections were incubated with 1% phloroglucinol-HCl (*v*/*v*) for 5 min and then observed under Zeiss optical microscope (Zeiss, Oberkochen, Germany).

Scanning electron microscopy (SEM) was used to observe cell wall structures and the effects of pretreatment. Cross sections were observed by SEM (PhenomtmPure FEI, USA) following the manual’s recommendations and images were captured digitally. The cell wall thickness in xylem fiber cells were measured using the software Image J (NIH, USA). For plant tissue in situ enzymatic digestion, the stem cross sections were pretreated with 1% NaOH as described below, washed with distilled water until pH 7.0 and incubated with 1 g/L mixed cellulase for 2 h at 50 °C. After enzymatic hydrolysis, the tissue samples were sputtered with gold and observed. For the effects of enzymatic digestion in vitro, the biomass residues after NaOH pretreatment and enzymatic digestion were dried to constant weight at 50 °C, and the surfaces of biomass samples were observed by SEM. Each sample was observed 10 times, and a representative image was used in this study.

#### Plant cell wall fractionation and determination

Plant cell wall fractionation and assay method were conducted as described previously [48] with minor modification, all experiments were performed in the technical triplicates. The stems of five plants were harvested, dried at 50–55 °C. The dried samples were powdered by passing through a 40-mesh screen and were stored in a dry container until use. Consecutive extractions with phosphate buffer, chloroform–methanol, dimethyl sulfoxide/water, and ammonium oxalate to remove soluble sugars, lipid, starch and pectin. Then the crude cell walls were used to collect the KOH-extractable hemicelluloses fraction. The remaining pellets were dissolved in 72% H_2_SO_4_ (*w*/*w*), and the supernatants were collected to determine hexose as cellulose level. Total hemicelluloses were calculated by measuring hexose and pentose of the hemicellulose fraction and the pentose of the remained cellulose pellets. Total lignin content was determined by two-step acid hydrolysis method as described. The crude cell wall samples were hydrolyzed with 67% H_2_SO_4_ (*v*/*v*) at 25 ℃ for 90 min with a gentle shaking at 150 rpm, and subsequently diluted to 3.97% (*w*/*w*) with distilled water and heated at 25 ℃ for 60 min. The acid-soluble lignin was solubilized during the hydrolysis process, and was measured by UV spectroscopy at 205 nm. The remaining residues were placed in a muffle furnace at 575 ± 25 ℃ for 4 h for the acid-insoluble lignin assay.

#### 2D-NMR assay of lignin composition and linkage

The NMR experiment was performed on a Bruker AVIII 400 MHz spectrometer as described [15, 46, 47]. The stem powders were incubated in acetate buffer (0.05 mM, 250 mL, pH = 4.8) with the loading of 6.0 mL Cel-lic^®^CTec2 (100 filter paper activity mL^−1^). Then, the reaction mixtures were incubated at 50 ℃ in a rotary shaker (50 g) for 2 days. After hydrolysis, the mixtures were centrifuged (2500 g), and the residual samples were washed with acetate buffer (0.05 mM, 250 mL, pH = 4.8) and freeze-dried with a smart freeze-dryer. The dry samples then underwent ball-milling for 120 min. The residual lignin was enzymatic hydrolyzed again in the same procedure. After the purification and drying process, the last samples were obtained. The chemical shifts of NMR spectra were calibrated with reference to dimethylsulfoxide (DMSO), used as an internal standard.

#### Chemical pretreatment and biomass enzymatic hydrolysis

Chemical pretreatment and sequential enzymatic hydrolysis were performed as described previously [48]. Pretreatment: the well-mixed biomass samples were treated with 6 mL H_2_SO_4_ under various concentrations (4%, *v*/*v*) at 120 °C for 20 min, then shaken under 150 rpm at 50 °C for 2 h. For NaOH pretreatment: the well-mixed biomass samples were incubated with 6 mL NaOH under various concentrations (4%, *w*/*v*) shaken at 50 °C for 2 h. For CaO pretreatment, the well-mixed biomass samples were treated with CaO at various concentrations (10%, *w*/*w*) shaken at 50 °C for 48 h. After pretreatments, the pretreated residues were washed with distilled water 5 times following enzymatic hydrolysis. Enzymatic hydrolysis: the pretreated biomass residues were washed with mixed-cellulase reaction buffer (0.2 M acetic acid–sodium acetate, pH 4.8), then incubated with 6 mL (1.6 g/L) of mixed-cellulases at 13.25 FPU/g biomass and xylanase at 8.40 U/g biomass (Imperial Jade Biotechnology Co., Ltd. Ningxia 750002, China), co-supplied with 1% Tween-80. The sealed samples were shaken under 150 rpm for 60 h at 50 °C. After centrifugation at 3000 *g* for 5 min, the supernatants were collected for pentoses and hexoses assay. All experiments were performed using five representative plants in triplicate.

#### Yeast fermentation and ethanol measurement

The yeast fermentation was conducted using *Saccharomyces cerevisiae* strain (Angel yeast Co., Ltd., Yichang, China) as previously described [48, 49]. The activated yeast (dissolved in 0.2 M phosphate buffer, pH 4.8) was inoculated into the mixture of enzymatic hydrolysates and residues with initial cell mass concentration at 0.5 g/L. The fermentation experiments were performed at 37 °C for 48 h, then distilled for determination of ethanol content. Ethanol content was measured using the dichromate oxidation method [48, 49]. All experiments were performed using five representative plants in triplicate.

#### Detection of cellulose features (CrI, DP)

The lignocellulose crystalline index (CrI) was detected with crude cell wall materials as described by Fan et al. [48]. Essentially, crystalline cellulose was extracted using 4 M KOH (containing 1.0 mg/mL sodium borohydride) followed by 8% (*w*/*v*) NaClO_2_ with 1.5% acetic acid at 25 °C for 72 h. The pellet was washed to neutral and dried before examination with X-ray diffraction (XRD) using Rigaku-D/MAX instrument (Uitima III, Japan). The biomass powder was laid on the glass sample holder (35 × 50 × 5 mm) and detected under plateau conditions. Ni-filtered Cu Kα radiation (*λ* = 0.154056 nm) generated at voltage of 40 kV and current of 18 mA, and scanned at speed of 0.0197°/s from 10 to 45 °C. The CrI was estimated using the intensity of the 200 peak (*I*_200_, *θ* = 22.5°) and the intensity at the minimum between the 200 and 110 peaks (*I*_am_, *θ* = 18.5°) as the follow: CrI = 100 × (*I*_200_− *I*_am_)/*I*_200_. Standard error of the CrI method was detected using five representative samples in triplicate.

The crude cellulose DP assay was performed using viscosity method as previously described according to the equation: DP^0.905^ = 0.75 [*η*]. And [η] is the intrinsic viscosity of the solution. All experiments were performed at 25 ± 0.5 °C using an Ubbelohde viscosity meter and cupriethylenediamine hydroxide (Cuen) as the solvent. The intrinsic viscosity was calculated by interpolation using the USP table (USP, 2002) that lists the predetermined values of the product of intrinsic viscosity and concentration. The [*η*] for cellulose samples exhibiting relative viscosity (*η*_re_) values between 1.1 and 9.9. *η*_rel_, was calculated using the equation: *η*_rel_ = *t*/*t*_0_, where t and *t*_0_ are the efflux times for the cellulose solution and Cuen (blank) solvent, respectively. Standard error of the DP method was detected using five representative samples in triplicate.

#### Crude cellulose hydrolysis by β-1,4-exoglucanase (CBHI)

CBHI enzyme hydrolysis assay was performed using crude cellulose samples as described by Huang et al. [50, 55]. Samples were incubated with CBHI (E.C. 3.2.1.91; Megazyme, USA) at 50 °C for a time course of reactions. After centrifugation, the supernatants were collected and treated with TFA, and *Myo*-inositol was added as the internal standard. The supernatants were then dried under vacuum to remove TFA. Distilled water and freshly prepared solution of sodium borohydride were added to each sample, incubated at 40 °C for 1 h, and the excess sodium borohydride was decomposed by adding acetic acid. 1-Methylimidazole and the acetic anhydride were added and mixed well to perform an acetylation reaction. The excess acetic anhydride was decomposed by adding distilled water. Dichloromethane was added, mixed gently, and left standing for phase separation. The collected samples were analyzed using GC–MS (SHIMADZU GCMS-QP2010 Plus) as previously described [50]. GC–MS Analytical Conditions: Restek Rxi-5 ms, 30 m × 0.25 mm ID × 0.25μm *df* column. Carrier gas: He. Injection method: split. Injection port: 250 °C, interface: 250 °C. Injection volume: 1.0 μL. The temperature program: from 170 °C (held for12 min) to 220 °C (held for 8 min) at 3 °C/min. Ion source temperature: 200 °C, ACQ Mode: SIM. The mass spectrometer was operated in the EI mode with ionization energy of 70 eV. Mass spectra were acquired with full scans based on the temperature program from 50 to 500 *m*/*z* in 0.45 s.

### Measurement of cellulose accessibility

Congo Red stain was applied to estimate cellulosic surface area accessible for degrading cellulases as previously described by Wiman et al. [56]. 100 mg sample was treated with Congo Red solution under increasing concentrations (0.25, 0.50, 0.75, 1.0, 1.5, 2.0 mg/mL) in 0.3 M phosphate buffer (pH 6.0) with 1.4 mM NaCl at 60 ºC for 24 h with 200 rpm rotation speed. After centrifugation at 8000*g* for 5 min, the absorbance of the supernatant was recorded at 498 nm. Adsorption of Congo Red (Ae, mg/g) was calculated by Langmuir model using the following equation: Ae = (Ci − Ce) × V/(M × 1000). V, total volume (mL) at determination; M, initial weight of biomass (g). In this study, V was 10 mL, M was 0.1 g. Ci and Ce, Congo Red concentrations (mg/L) before or after adsorption, calculated using standard curve from Congo Red solutions at 20, 40, 60, 80, 100 and 120 mg/L concentrations.

### Statistical analysis

Biological triplicate samples were collected for 5 plants of each transgenic line selection, and chemical analysis was performed in technical triplicates. The SPSS statistical software was used for data analysis. Statistical analysis was performed by Student’s *t*-tests (two-tail distribution and two samples with unequal variances) as *P < 0.05 and **P < 0.01.

## Supplementary Information

**Additional file 1:** **Table S1. **Gene primers used in this study. **Figure S1. **Measurement of plant growth and gene expression in transgenic poplar plants. (a) Images of 3-month-old transgenic poplar lines and wild type (WT); Scale bar as 10 cm. (b) Expression of cell differentiation genes in *PtoMYB115 *transgenic plants and WT. Primers are listed in Table S1. The poplar *ubiquitin *gene was used as an internal control. All data are given as means ± SD from three biological repeats. Statistical analyses were performed using Student’s *t *test as **P < 0.01 (n = 3). **Figure S2. **Observations of plant cell wall formation in the *PtoMYB115 *transgenic lines and WT. (a) Cell wall thickness of SEM observation; (b) Cellulose and hemicellulose contents (% biomass). All data as means ± SD. Student’s *t*-test was performed between the transgenic line and WT as **P < 0.01 (n = 3). **Figure S3. **Comparison of lignocellulose features between the transgenic lines and WT. (a) Crystalline index (CrI) of crude cellulose. (b) Correlation analysis between lignocellulose features and hexose yields (% biomass) released from enzymatic hydrolyses after H_2_SO_4_ or CaO pretreatment. **Significant correlation at *P *< 0.01 (n = 15). **Figure S4. **Correlation analysis between DP of cellulose and hexose yields (% biomass) released from enzymatic hydrolyses after pretreatments. **Figure S5. **Quantitative RT-PCR analysis of proanthocyanidin biosynthetic genes in the *PtoMYB115 *transgenic lines and WT.

## Data Availability

All data generated or analyzed during this study are included in this published article and its additional file. Plant materials used in this study are available from corresponding author, Keming Luo (kemingl@swu.edu.cn).

## Abbreviations

Xy: Xylem
Xf: Xylem fiber cells
Xv: Xylem vessel cells
SEM: Scanning electron microscopy
CrI: Cellulose crystallinity index
DP: Degree of polymerization
CBH: Cellobiohydrolase
