# Supplementary material for: Overexpression of PtoMYB115 improves lignocellulose recalcitrance to enhance biomass digestibility and bioethanol yield by specifically regulating lignin biosynthesis in transgenic poplar
Source: Biotechnol Biofuels Bioprod. 2022 Nov 5;15:119. doi: 10.1186/s13068-022-02218-7 (PMC9636778; doi:10.1186/s13068-022-02218-7)
Supplement: Supplementary file 1 — Additional file 1: Table S1. Gene primers used in this study. Figure S1. Measurement of plant growth and gene expression in transgenic poplar plants. (a) Images of 3-month-old transgenic poplar lines and wild type (WT); Scale bar as 10 cm. (b) Expression of cell differentiation genes in PtoMYB115 transgenic plants and WT. Primers are listed in Table S1. The poplar ubiquitin gene was used as an internal control. All data are given as means ± SD from three biological repeats. Statistical analyses were performed using Student’s t test as **P < 0.01 (n = 3). Figure S2. Observations of plant cell wall formation in the PtoMYB115 transgenic lines and WT. (a) Cell wall thickness of SEM observation; (b) Cellulose and hemicellulose contents (% biomass). All data as means ± SD. Student’s t-test was performed between the transgenic line and WT as **P < 0.01 (n = 3). Figure S3. Comparison of lignocellulose features between the transgenic lines and WT. (a) Crystalline index (CrI) of crude cellulose. (b) Correlation analysis between lignocellulose features and hexose yields (% biomass) released from enzymatic hydrolyses after H2SO4 or CaO pretreatment. **Significant correlation at P < 0.01 (n = 15). Figure S4. Correlation analysis between DP of cellulose and hexose yields (% biomass) released from enzymatic hydrolyses after pretreatments. Figure S5. Quantitative RT-PCR analysis of proanthocyanidin biosynthetic genes in the PtoMYB115 transgenic lines and WT. [file 13068_2022_2218_MOESM1_ESM.pptx]

## Slide 1
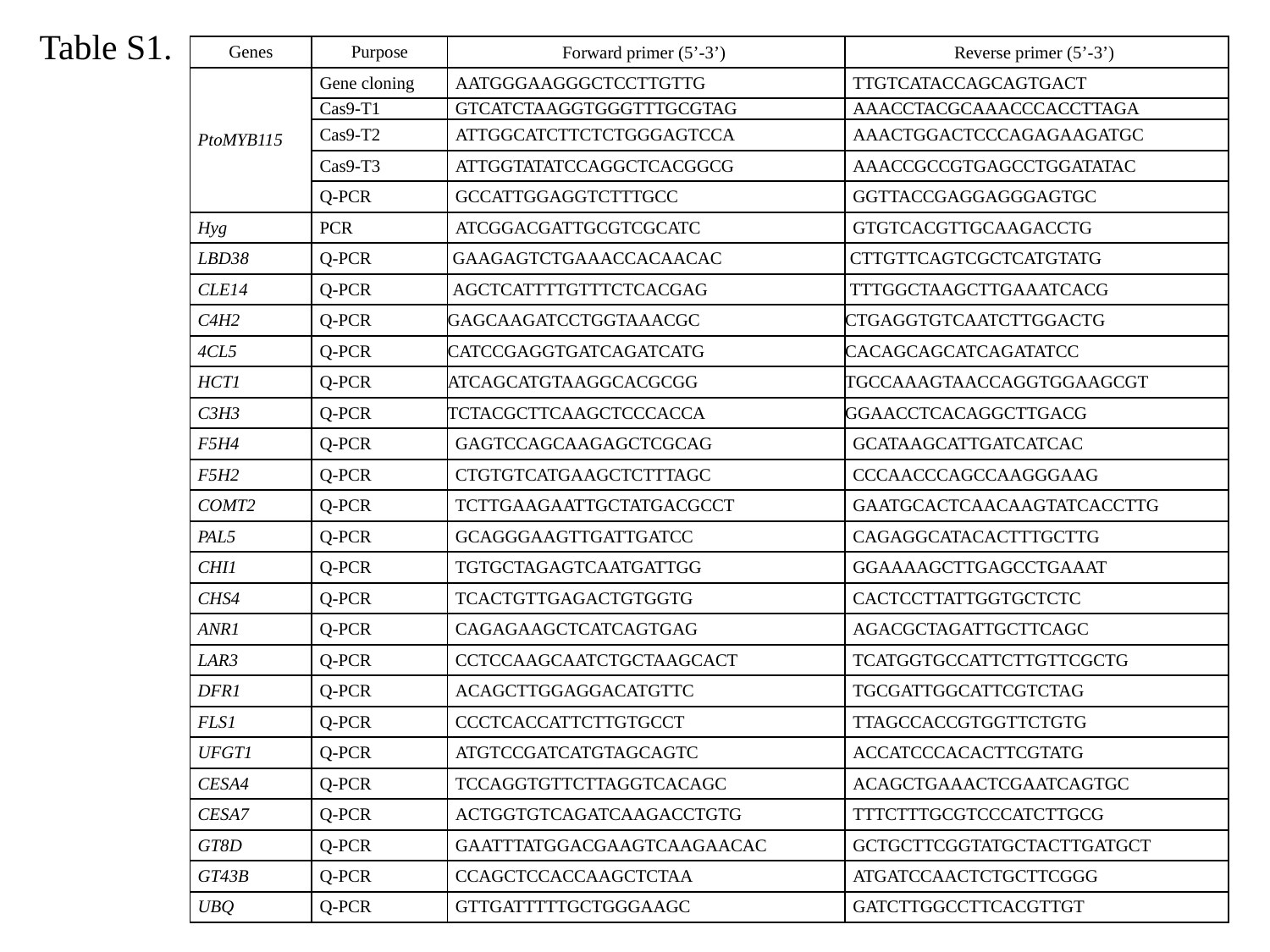

# Table S1.
| Genes | Purpose | Forward primer (5’-3’) | Reverse primer (5’-3’) |
| --- | --- | --- | --- |
| PtoMYB115 | Gene cloning | AATGGGAAGGGCTCCTTGTTG | TTGTCATACCAGCAGTGACT |
| | Cas9-T1 | GTCATCTAAGGTGGGTTTGCGTAG | AAACCTACGCAAACCCACCTTAGA |
| | Cas9-T2 | ATTGGCATCTTCTCTGGGAGTCCA | AAACTGGACTCCCAGAGAAGATGC |
| | Cas9-T3 | ATTGGTATATCCAGGCTCACGGCG | AAACCGCCGTGAGCCTGGATATAC |
| | Q-PCR | GCCATTGGAGGTCTTTGCC | GGTTACCGAGGAGGGAGTGC |
| Hyg | PCR | ATCGGACGATTGCGTCGCATC | GTGTCACGTTGCAAGACCTG |
| LBD38 | Q-PCR | GAAGAGTCTGAAACCACAACAC | CTTGTTCAGTCGCTCATGTATG |
| CLE14 | Q-PCR | AGCTCATTTTGTTTCTCACGAG | TTTGGCTAAGCTTGAAATCACG |
| C4H2 | Q-PCR | GAGCAAGATCCTGGTAAACGC | CTGAGGTGTCAATCTTGGACTG |
| 4CL5 | Q-PCR | CATCCGAGGTGATCAGATCATG | CACAGCAGCATCAGATATCC |
| HCT1 | Q-PCR | ATCAGCATGTAAGGCACGCGG | TGCCAAAGTAACCAGGTGGAAGCGT |
| C3H3 | Q-PCR | TCTACGCTTCAAGCTCCCACCA | GGAACCTCACAGGCTTGACG |
| F5H4 | Q-PCR | GAGTCCAGCAAGAGCTCGCAG | GCATAAGCATTGATCATCAC |
| F5H2 | Q-PCR | CTGTGTCATGAAGCTCTTTAGC | CCCAACCCAGCCAAGGGAAG |
| COMT2 | Q-PCR | TCTTGAAGAATTGCTATGACGCCT | GAATGCACTCAACAAGTATCACCTTG |
| PAL5 | Q-PCR | GCAGGGAAGTTGATTGATCC | CAGAGGCATACACTTTGCTTG |
| CHI1 | Q-PCR | TGTGCTAGAGTCAATGATTGG | GGAAAAGCTTGAGCCTGAAAT |
| CHS4 | Q-PCR | TCACTGTTGAGACTGTGGTG | CACTCCTTATTGGTGCTCTC |
| ANR1 | Q-PCR | CAGAGAAGCTCATCAGTGAG | AGACGCTAGATTGCTTCAGC |
| LAR3 | Q-PCR | CCTCCAAGCAATCTGCTAAGCACT | TCATGGTGCCATTCTTGTTCGCTG |
| DFR1 | Q-PCR | ACAGCTTGGAGGACATGTTC | TGCGATTGGCATTCGTCTAG |
| FLS1 | Q-PCR | CCCTCACCATTCTTGTGCCT | TTAGCCACCGTGGTTCTGTG |
| UFGT1 | Q-PCR | ATGTCCGATCATGTAGCAGTC | ACCATCCCACACTTCGTATG |
| CESA4 | Q-PCR | TCCAGGTGTTCTTAGGTCACAGC | ACAGCTGAAACTCGAATCAGTGC |
| CESA7 | Q-PCR | ACTGGTGTCAGATCAAGACCTGTG | TTTCTTTGCGTCCCATCTTGCG |
| GT8D | Q-PCR | GAATTTATGGACGAAGTCAAGAACAC | GCTGCTTCGGTATGCTACTTGATGCT |
| GT43B | Q-PCR | CCAGCTCCACCAAGCTCTAA | ATGATCCAACTCTGCTTCGGG |
| UBQ | Q-PCR | GTTGATTTTTGCTGGGAAGC | GATCTTGGCCTTCACGTTGT |

## Slide 2
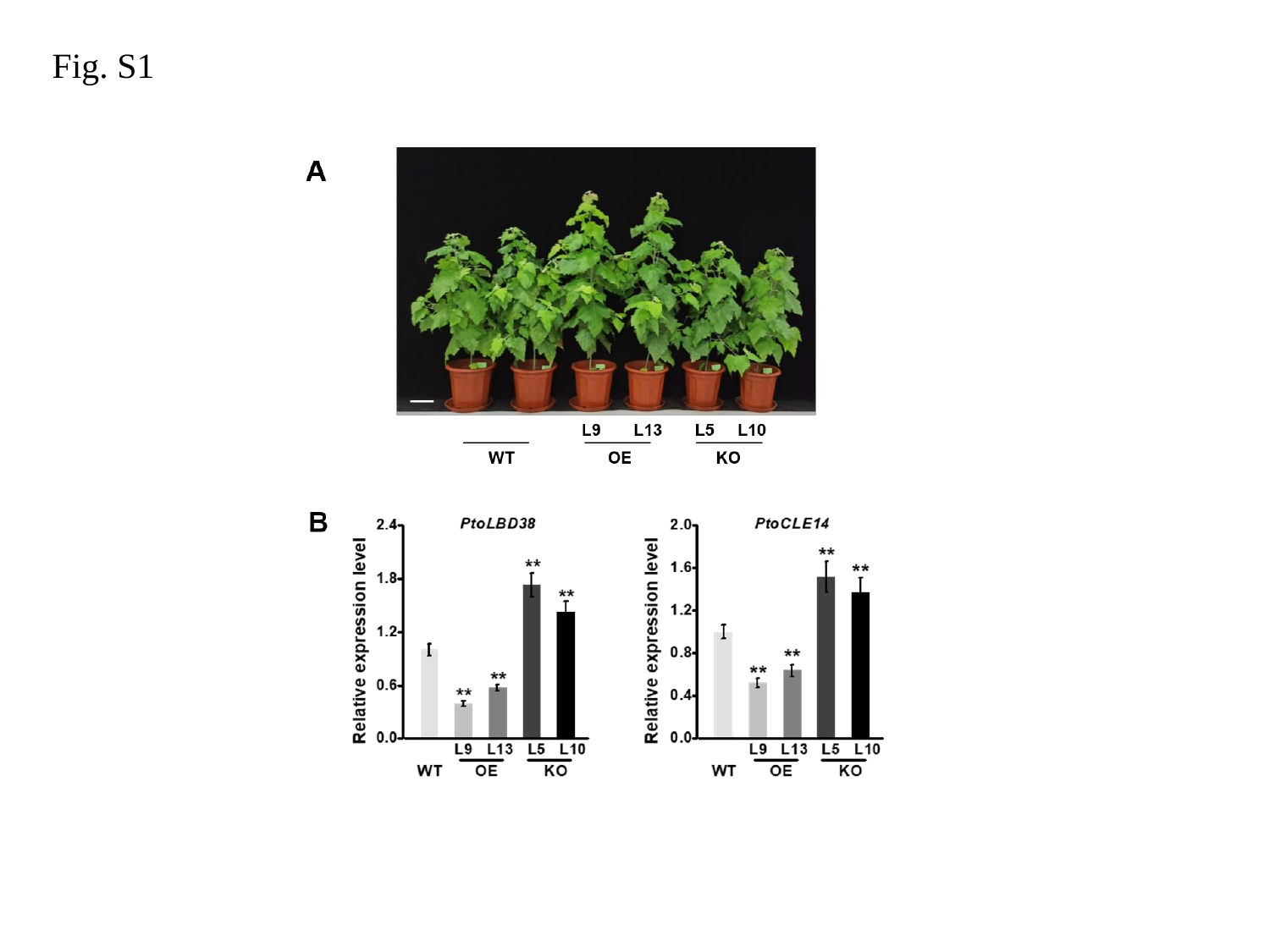

Fig. S1

## Slide 3
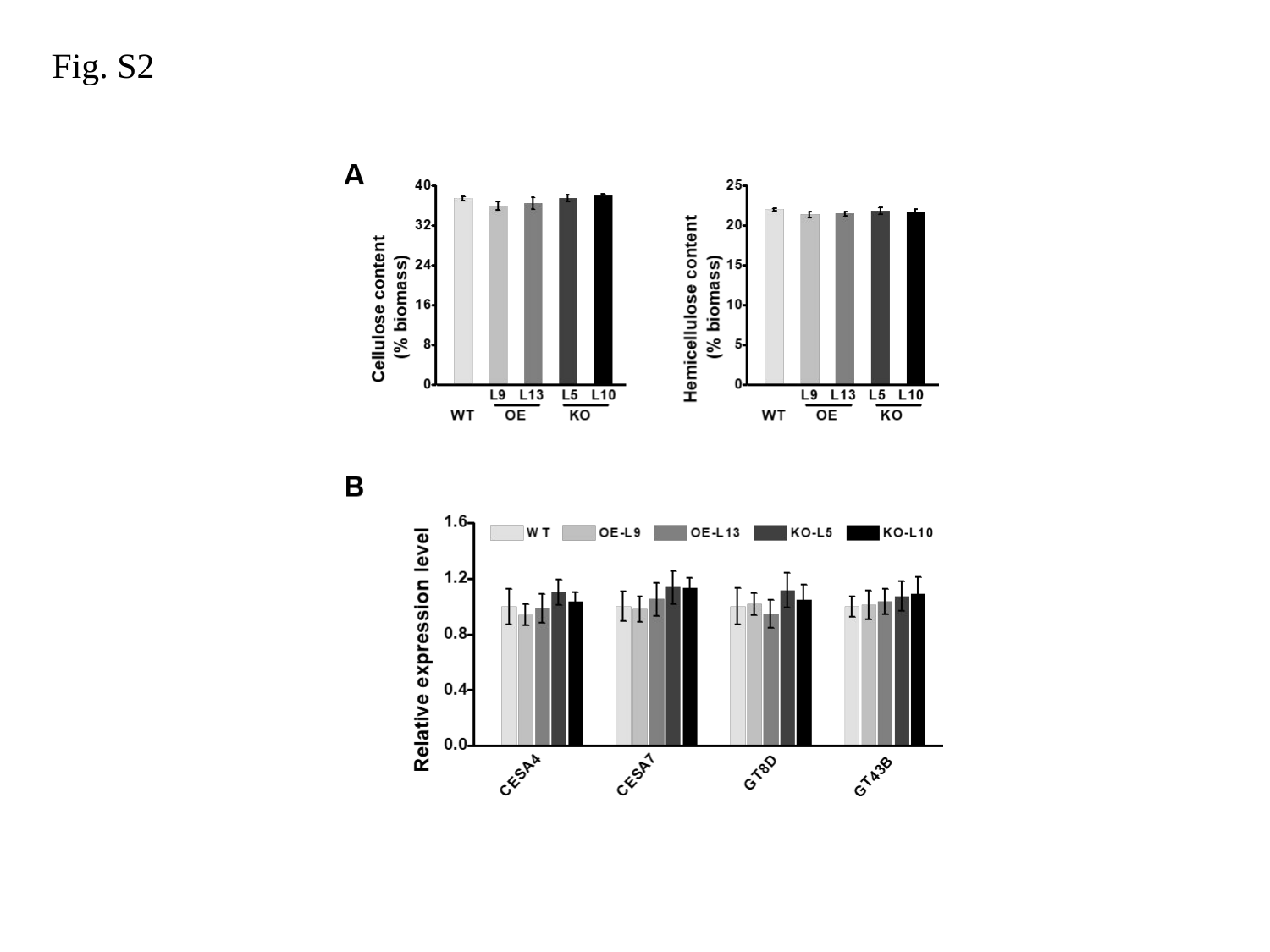

Fig. S2

## Slide 4
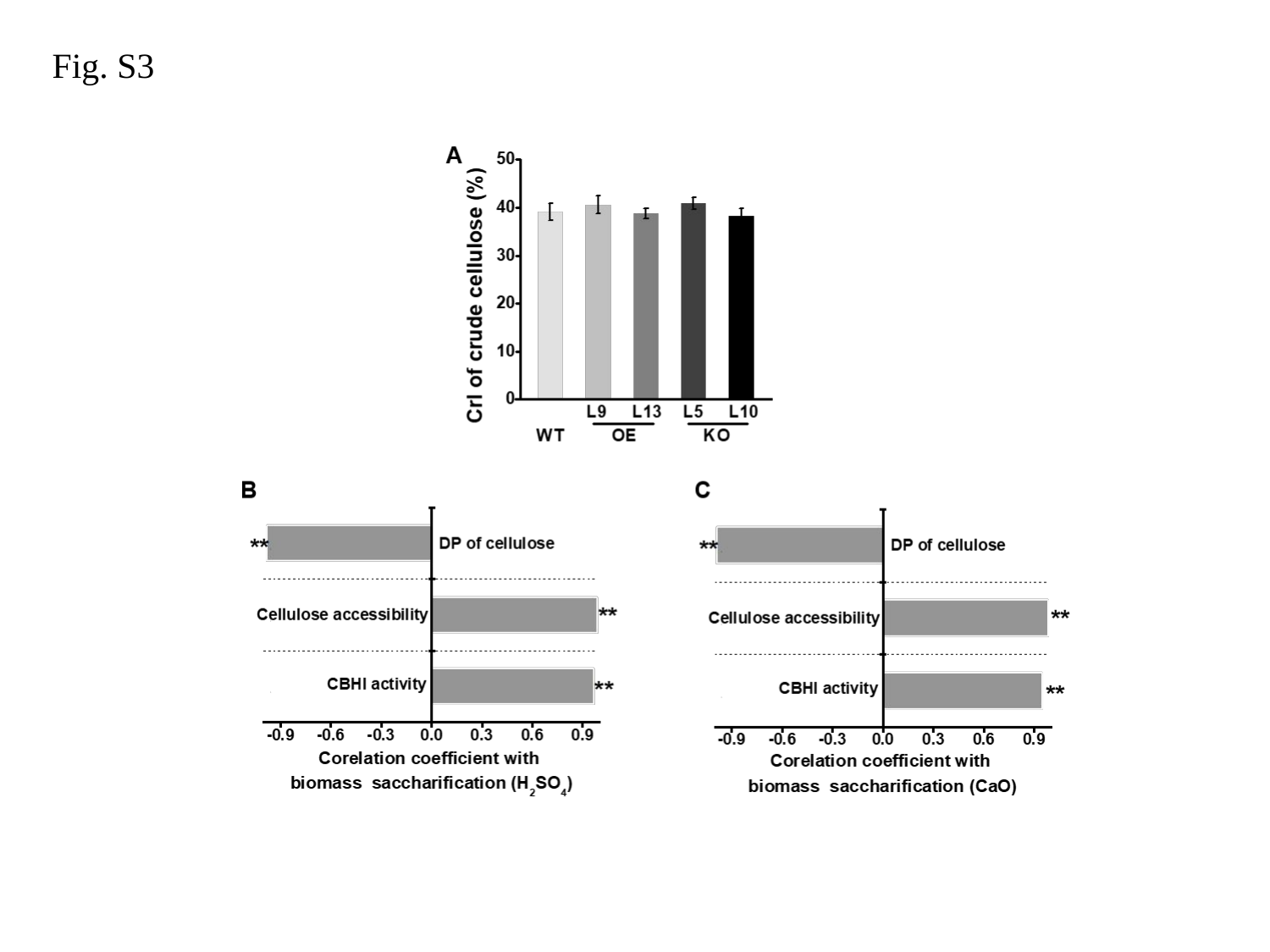

Fig. S3

## Slide 5
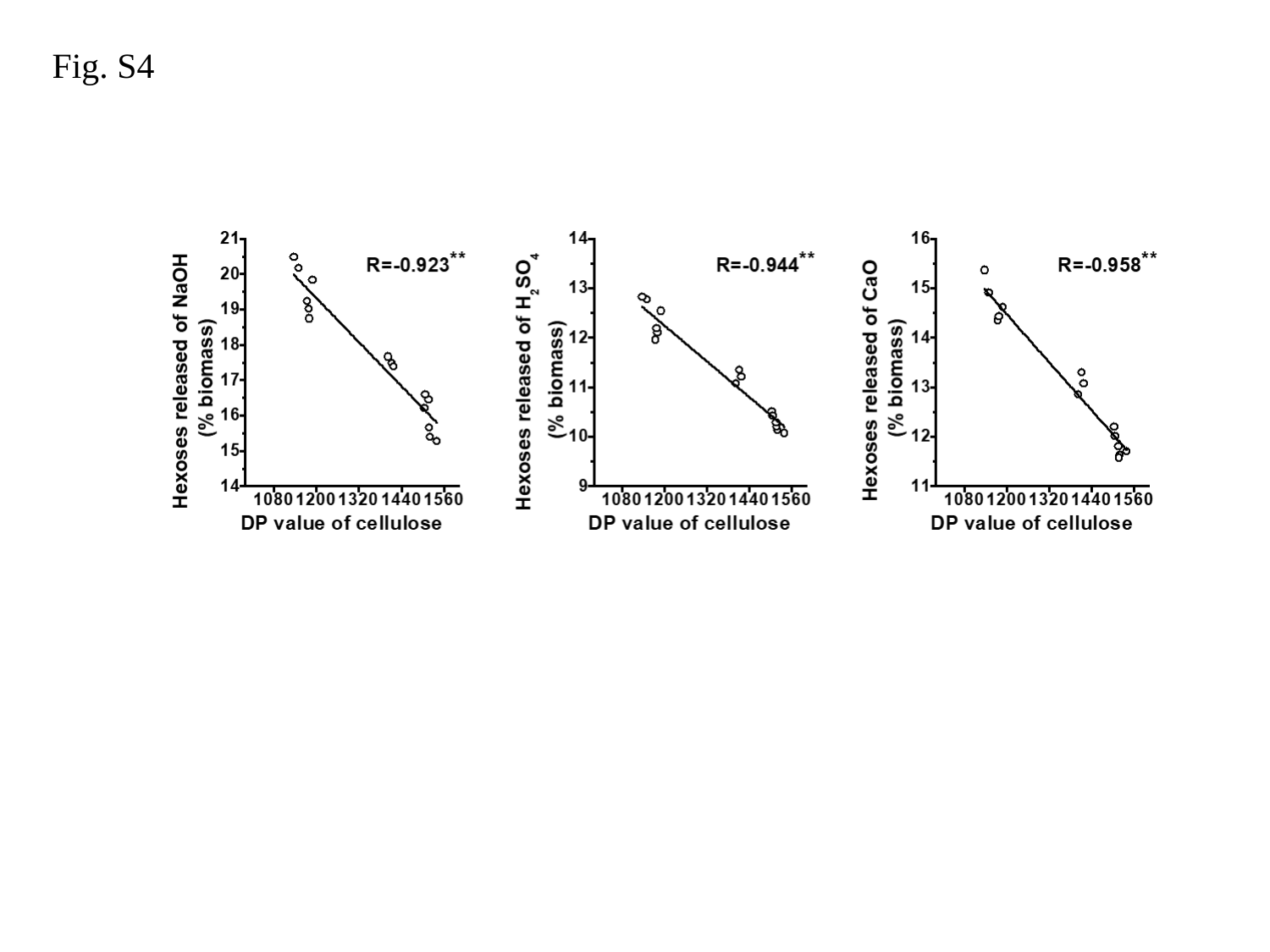

Fig. S4

## Slide 6
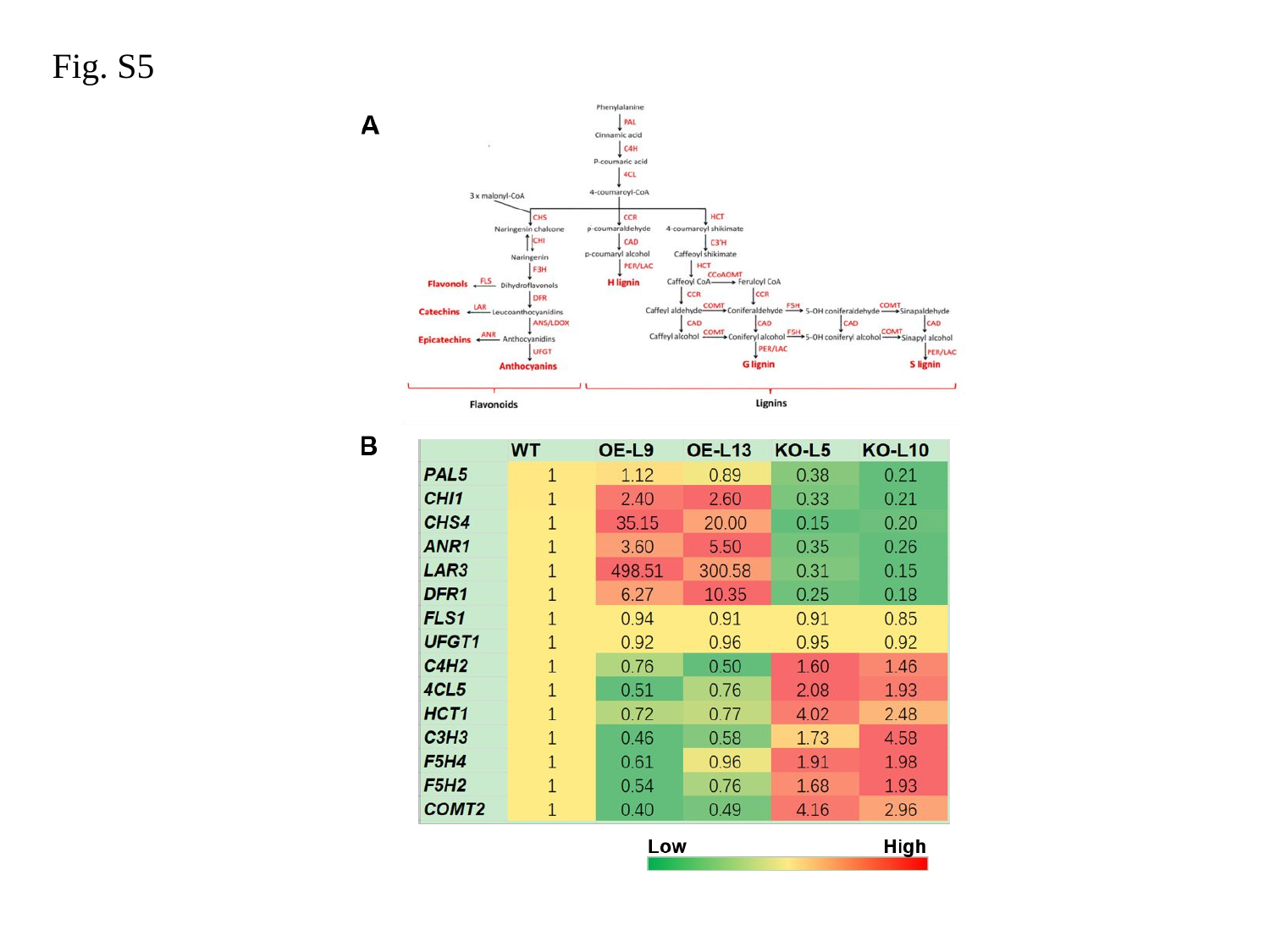

Fig. S5
